# Anvirzel™ in combination with cisplatin in breast, colon, lung, prostate, melanoma and pancreatic cancer cell lines

**DOI:** 10.1186/2050-6511-14-18

**Published:** 2013-03-25

**Authors:** Panagiotis Apostolou, Maria Toloudi, Marina Chatziioannou, Eleni Ioannou, Dennis R Knocke, Joe Nester, Dimitrios Komiotis, Ioannis Papasotiriou

**Affiliations:** 1 Research Genetic Cancer Centre Ltd (R.G.C.C. Ltd), Filotas, Florina, Greece; 2 Nerium Biotechnology, Inc. San Antonio, Texas, USA; 3 Department of Biochemistry & Biotechnology, University of Thessaly, Larisa, Greece

**Keywords:** Anvirzel™, Cisplatin, Viability assays, Cancer cell lines, Methyl-tetrazolium dye

## Abstract

**Background:**

Platinum derivatives are used widely for the treatment of many cancers. However, the toxicity that is observed makes imperative the need for new drugs, or new combinations. Anvirzel™ is an extract which has been demonstrated with experimental data that displays anticancer activity. The aim of the present study is to determine whether the combination of Cisplatin and Anvirzel™ has a synergistic effect against different types of cancer.

**Materials and methods:**

To measure the efficacy of treatment with Cisplatin and Anvirzel™, methyl-tetrazolium dye (MTT) chemosensitivity assays were used incorporating established human cancer cell lines. Measurements were performed in triplicates, three times, using different incubation times and different concentrations of the two formulations in combination or on their own. t-test was used for statistical analysis.

**Results:**

In the majority of the cell lines tested, lower concentrations of Anvirzel™ induced a synergistic effect when combined with low concentrations of Cisplatin after an incubation period of 48 to 72 h. The combination of Anvirzel™/Cisplatin showed anti-proliferative effects against a wide range of tumours.

**Conclusion:**

The results showed that the combination of Anvirzel™ and Cisplatin is more effective than monotherapy, even when administered at low concentrations; thus, undesirable toxic effects can be avoided.

## Background

Many studies demonstrate the anti-proliferative activity of Oleandrin. These properties make it attractive for use as a treatment for cancer [1-5]; however, a major problem is that Oleandrin is toxic to normal cells and tissues [6,7]. Anvirzel™ is an extract of *Nerium oleander* comprised primarily of Oleandrin and Oleandrigenin [8]. Recent studies demonstrate that Anvirzel™ decreases viability in prostate cancer cell lines as well as a wide range of other human cancer cell lines [9-12]. Cisplatin (CDDP) is a platinum-based chemotherapy drug used to treat various types of cancer [13,14]. Because it is highly toxic, and because of primary and secondary resistance of cancer cells to Cisplatin [15], it is commonly used in combination with other drugs [16,17]. A recent study reported that the combination of Anvirzel™, Carboplatin and Docetaxel is more effective than monotherapy [18]. Therefore, the aim of the current study was to determine whether the combination of Anvirzel™ and Cisplatin was more effective than the use of either drug alone using MTT chemosensitivity assays based on human cancer cell lines [19-23].

## Methods

The human carcinoma cell lines used were obtained from the ECACC-HPA (European Collection of Cell Cultures - Health Protective Agency, UK). PC3, LNCaP and 22Rv1 are human prostate cancer cell lines, MDA-MB 231, T47D, and MCF-7 are human breast cancer lines, CALU-1, COLO699N and COR-L 105 are non-small cell lung carcinoma lines (NSCLC), HCT-116, HT55 and HCT-15 are colorectal cancer lines, and A375 and PANC-1 are melanoma and pancreatic cancer cells lines, respectively. MTT chemosensitivity assays were used to determine the efficacy of combined treatment compared with that each drug alone. The incubation times used in the study were 24, 48 and 72 h at concentrations ranging from 0.01 ng/ml to 10 ng/ml for Anvirzel™ and from 0.1 μg/ml to 100 μg/ml for Cisplatin.

### Cell lines

Cells were cultured in 75 cm^2^ flasks (Orange Scientific, 5520200) in the recommended media supplemented with the appropriate amount of heat inactivated Fetal Bovine Serum (FBS, Invitrogen, 10106–169, California) and 2 mM L-Glutamine (Sigma, G5792, Germany). The cells were maintained at 37°C in a 5% CO_2_ atmosphere.

### Viability assays

Cells were detached by trypsinisation (Trypsin-0.25% EDTA, Invitrogen, 25200–072) during the logarithmic phase of growth and plated in 96-well plates (Corning, Costar 3595) at a density of 18,000 cells/well in a final volume of 200 μl medium per well. When the cells reached 70–80% confluence, the medium was removed and Anvirzel™ (Salud Integral; diluted in water) and Cisplatin (Sigma, P4394; diluted in N, N-dimethylformamide; Fluka, 40255) were added to the cells at different concentrations. Absorbance was measured after 24, 48 and 72 h of incubation.

#### MTT assay

For the MTT assay, methyl-tetrazolium dye (Sigma, M2128) was added to each well at a concentration of 5 mg/ml (diluted in PBS) and the plates incubated for 3 h at 37°C. The medium was then discarded and the cells rinsed with PBS. Finally, the formazan crystals were dissolved in dimethylsulphoxide (Sigma, D4540).

To calculate the fold-decrease in staining the absorbance was calculated using the Beer-Lambert law: A = εcl, where “A” is the absorbance, “ε” is an extinction coefficient, “l” is the distance the light travels through the material, and “c” is the concentration of the absorbing species [24].

The optical density of the plate was measured using a μQuant spectrophotometer (μQuant Biomolecular Spectrophotometer MQX200) and the data analysed with Gen5 software (Gen5™ Microplate Data Collection & Analysis software, BioTek® Instruments. Inc, April 2008). Absorbance was measured at 570 nm and a second wavelength at 630 nm was measured to subtract background “noise”.

### Statistical analysis

All treatments for each cell line were performed in triplicate, three times. The statistical significance of all effects was evaluated using the “difference of the means” test. A p value < 0.05 was considered significant.

## Results

**Figure 1:**
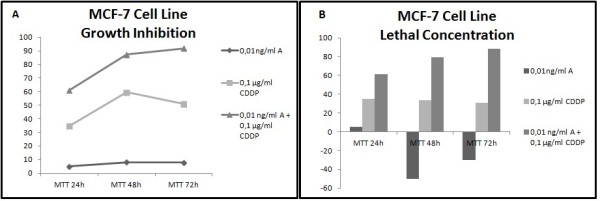
**A. The illustration shows the effect of Anvirzel™ and Cisplatin, and their combination compared with the growth rate of cells in cell line MCF-7 which represents breast cancer. B**. The effect of the above drugs in monotherapy as well their combination compared with the lethality of cells.

**Figure 2:**
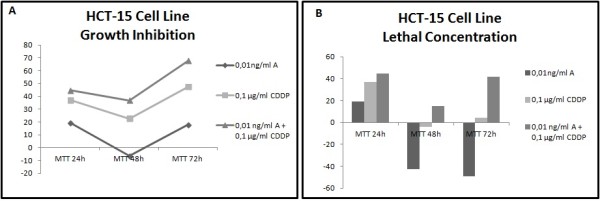
**A. The illustration shows the effect of Anvirzel™ and Cisplatin, and their combination compared with the growth rate of cells in cell line HCT-15 which represents colorectal cancer. B**. The effect of the above drugs in monotherapy as well their combination compared with the lethality of cells.

**Figure 3:**
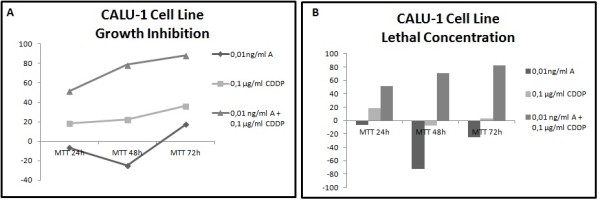
**A. The illustration shows the effect of Anvirzel™ and Cisplatin, and their combination compared with the growth rate of cells in cell line CALU-1 which represents NSCLC. B**. The effect of the above drugs in monotherapy as well their combination compared with the lethality of cells.

**Figure 4:**
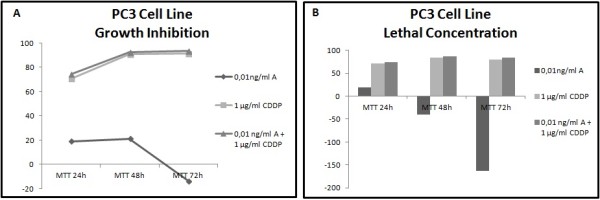
**A. The illustration shows the effect of Anvirzel™ and Cisplatin, and their combination compared with the growth rate of cells in cell line PC3 which represents prostate carcinoma. B**. The effect of the above drugs in monotherapy as well their combination compared with the lethality of cells.

**Figure 5:**
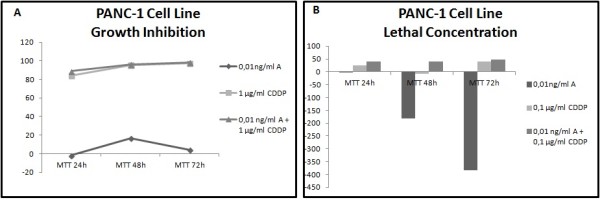
**A. The illustration shows the effect of Anvirzel™ and Cisplatin, and their combination compared with the growth rate of cells in cell line PANC-1 which represents pancreatic cancer. B**. The effect of the above drugs in monotherapy as well their combination compared with the lethality of cells.

**Figure 6:**
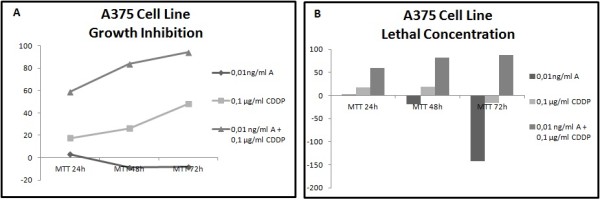
**A. The illustration shows the effect of Anvirzel™ and Cisplatin, and their combination compared with the growth rate of cells in cell line A375 which represents melanoma cancer. B**. The effect of the above drugs in monotherapy as well their combination compared with the lethality of cells.

Different results were observed not only between each type of cancer but also between each cell line within the same carcinoma group. In all cell lines has been studied LC50 (lethal concentration 50), which is the concentration that kill half of the sample population, and GI50 (growth inhibition 50), which is the concentration required to inhibit growth by 50%. In all cell lines tested, administration of the combined formulation at lower concentrations was more effective than monotherapy. The formulation that elicited the most effective results in most of the cell lines was a combination of 0.01 ng/ml Anvirzel™ and 0.1 μg/ml Cisplatin, with incubation period of 48 h or 72 h. The combination of 0.01 ng/ml Anvirzel™ and 1 μg/ml Cisplatin was more effective in pancreatic cancer cell line, as well in PC3 and MDA-MB231 cell lines. The Figures 1, 2, 3, 4, 5, 6 represent data from cell lines that were tested both for Growth inhibition and for Lethal Concentrations.

## Discussion

Anvirzel™ is an extract of *Nerium oleander* (family Apocynaceae) that contains two toxic cardiac glycosides, Oleandrin and Oleandrigenin [8], which have anti-proliferative effects against various types of cancer. According to literature data, *Nerium oleander* is often used for its healing properties [25]. Oleandrin is used to treat heart failure, and alters the levels of intracellular K^+^ and Ca^2+^[26]. Other studies show that Oleandrin suppresses the activation of many transcription factors and enhances the radiosensitivity of tumours [27,28]. Oleandrin induces cell death through the activation of caspases in a variety of human tumour cells, as well as by activating calcineurin and NF-AT via the Fas ligand [3]. Recent studies of Anvirzel™ in prostate cell lines show that it interacts with the membrane Na^+^/K^+^-ATPase and thus inhibits the export of FGF-2 [11,28]. Cisplatin is a platinum-based chemotherapy drug widely used to treat various types of cancer [13,14]. In high concentrations, Cisplatin is highly cytotoxic. In addition, tumour cells often develop resistance to treatment [15]. Many studies demonstrate that administration of Cisplatin in combination with other treatments is more effective and produces fewer toxic effects. The present study aimed the demonstrate the synergistic effect of Anvirzel™ and Cisplatin in a wide range of cell lines, which represent the most common types of cancer. It was used the methyl-tetrazolium dye assay, which is used to measure the activity of enzymes in the mitochondria. During this assay, the dye is taken up by endocytosis and reduced by the mitochondrial enzymes to yield formazan, which is purple/blue [19,20].

It has been confirmed that Anvirzel™ has better activity at lower concentrations in many cancer cell lines, after 48 or 72 h of incubation. In contrast, low Cisplatin concentrations (<1 μg/ml), do not indicate activity in the same cells. The effect of platinum is evident at concentrations greater than 10 μg/ml. However, these concentrations have high cytotoxicity effects. The combination of the two formulations at very low concentrations is able to inhibit cell growth by more than 50% in an exposure time of 72 hours. The same concentration may also reduce the number of live cells at rates up to 85% in the same time of exposure. Lowered effect observed in cell lines that are hormone-dependent, cases such as breast and prostate cancer.

## Conclusions

The MTT viability assays were used to test the hypothesis that combined treatment with Cisplatin and Anvirzel™ would be more effective than either drug alone. The results of all three assays were both concentration and cell line-dependent. It is noteworthy that increased efficacy was observed at lower concentrations of both substances, 0.01 ng/ml for Anvirzel™ and 0.1 μg/ml for Cisplatin, than of either drug alone. The results were neither reliable nor reproducible at higher concentrations of Anvirzel™ and Cisplatin. The results were also time-dependent: treatment was effective after 48 h and 72 h of incubation, but not after 24 h.

The present study contributes to demonstrate an effective interaction between Anvirzel™ and another widely used drug with cytostatic effects. Based on these data, it is crucial to perform further studies to identify and characterize the interaction between Anvirzel™ and other drugs currently used to treat cancer.

## Abbreviations

CDDP: Cisplatin; MTT: 3-(4,5-Dimethylthiazol-2-yl)-2,3-diphenyltetrazolium bromide; NSCLC: Non-small cell lung cancer

## Competing interests

The authors declare that they have no competing interests.

## Authors’ contributions

PA carried out the chemosensitivity assays, drafted the manuscript and performed the statistical analysis. MT participated in the chemosensitivity assays. MC participated in the chemosensitivity assays. EI carried out the cell lines culture. DK participated in the design of the study and coordination. JN participated in the design of the study and coordination. DK participated in the design of the study. IP supervised the assays and the manuscript. All authors read and approved the final manuscript.

## Pre-publication history

The pre-publication history for this paper can be accessed here:

http://www.biomedcentral.com/2050-6511/14/18/prepub
